# Comparing ventilation modes by electrical impedance segmentography in ventilated children

**DOI:** 10.1007/s10877-022-00828-y

**Published:** 2022-02-14

**Authors:** Jennifer Bettina Brandt, Alex Mahlknecht, Tobias Werther, Roman Ullrich, Michael Hermon

**Affiliations:** 1 Division of Neonatology, Medical University of Vienna, Pediatric Intensive Care & Neuropediatrics, Vienna, Austria; 2 Hospital of the Brothers of St. John of God, Eisenstadt, Austria; 3 Department for Anesthesia, Intensive Care Medicine and Pain Medicine, Medical University of Vienna, Vienna, Austria

**Keywords:** NAVA, Electrical Impedance Segmentography, Personalized ventilation, Bedside monitoring, Dependent lung area, Pediatric ventilation

## Abstract

Electrical impedance segmentography offers a new radiation-free possibility of continuous bedside ventilation monitoring. The aim of this study was to evaluate the efficacy and reproducibility of this bedside tool by comparing synchronized intermittent mandatory ventilation (SIMV) with neurally adjusted ventilatory assist (NAVA) in critically-ill children. In this prospective randomized case–control crossover trial in a pediatric intensive care unit of a tertiary center, including eight mechanically-ventilated children, four sequences of two different ventilation modes were consecutively applied. All children were randomized into two groups; starting on NAVA or SIMV. During ventilation, electric impedance segmentography measurements were recorded. The relative difference of vertical impedance between both ventilatory modes was measured (median 0.52, IQR 0–0.87). These differences in left apical lung segments were present during the first (median 0.58, IQR 0–0.89, p = 0.04) and second crossover (median 0.50, IQR 0–0.88, p = 0.05) as well as across total impedance (0.52 IQR 0–0.87; p = 0.002). During NAVA children showed a shift of impedance towards caudal lung segments, compared to SIMV. Electrical impedance segmentography enables dynamic monitoring of transthoracic impedance. The immediate benefit of personalized ventilatory strategies can be seen when using this simple-to-apply bedside tool for measuring lung impedance.

## Introduction

Patients at a pediatric intensive care unit (PICU) often require mechanical ventilation due to various diagnoses and medical interventions. Optimized strategies for patient-oriented mechanical ventilation are an important and frequently discussed topic in daily pediatric intensive care and scientific studies. Various ventilation modes have been established over the years, but have repeatedly shown undesirable side effects for children [1–4]. Synchronized intermittent mandatory ventilation (SIMV) has been shown to be a lung-protective strategy in pediatric intensive care [5]. Patient-ventilator-asynchrony during SIMV, has been described as pronounced [6] and even more in comparison to neurally adjusted ventilatory assist (NAVA) [7]. Due to this asynchrony increased doses of sedation in ventilated children are required as documented in a study by Baez Hernandez et al. [7]. Furthermore, several studies have described diaphragmatic atrophy caused by mechanical mandatory ventilation in pediatric patients [1–3].

NAVA, on the other hand, is triggered by patients’ diaphragmatic neural breathing effort itself [8], by the placement of a special nasogastric tube. The electrical activity of the diaphragm (Eadi) is monitored and used as a trigger to induce assisted ventilatory support. NAVA varies its support according to the signals of the diaphragm and the level of pressure during ventilation. This triggering mechanism enables improved patient-ventilator-synchrony [8, 9] and therefore reduces the need for sedation [7, 10]. This is presumably also associated with a greater extubation success [11]. According to a recent study, children who underwent cardiac surgery also had lower positive inspiratory pressure (PIP) levels on NAVA compared to children on SIMV [7]. To assess different forms of ventilation strategies used for children, various radiation-free imaging technologies such as lung ultrasound [12], electrical impedance tomography (EIT) [13] and segmentography (EIS) [14] have been applied in studies as well as in clinical routine. Electric impedance has been used as a monitoring tool for mechanical ventilation in infants with ARDS, prematurely born children [15], those with bronchiolitis [16], and in children after cardiac surgery [17]. For continuous ventilatory monitoring EIT utilizes 16 to 32 electrodes, that are mounted around the circumference of the thorax and provide real-time imaging of tissue composition via simultaneous injection and measurement of electrical currents. EIS, on the other hand, only uses 10 electrodes applied on the frontal and dorsal plane of the thorax, imaging a tissue composition in four regions (upper and lower; left and right region of the lung), from which potential vertical (between both, upper and lower lung regions) and horizontal (between right and left lung regions) impedance shift can be extrapolated. Measurements of impedance therefore enable clinicians to draw conclusions about global and regional ventilation of the lung in spontaneously breathing as well as in mechanically-ventilated children [13, 18].

Since segmentography of lung impedance is a relatively new imaging method in pediatrics, the aim of this study was to evaluate the efficacy and reproducibility of this bedside tool by performing continuous measurements during pressure-controlled and breath-supported mandatory ventilation (SIMV (PC) PS) compared to NAVA in critically-ill children.

## Materials and methods

### Setting

After obtaining the approval of the ethics committee of the Medical University of Vienna (MUV, EK No 1668/2018) we performed a prospective single center randomized crossover trial at the Department of Pediatrics and Adolescent Medicine. This trial was conducted from April 2019 to June 2020 at the neonatal and pediatric intensive care unit. Written informed consent of all parents or legal guardians was obtained prior to the start and randomization of the study groups.

### Patients

Children up to 12 months of age, mechanically ventilated and hemodynamically stable in the preceding 24 h of the intervention, were included in our study. Children with phrenic palsy or on muscle relaxants were excluded from EIS measurements. Ventilation was performed with a Servo-u ventilator (Maquet Critical Care, Solna, Sweden). All children included had suitable nasogastric tubes for performing NAVA, according to clinical indications made by independent physicians of the PICU prior to the study. This study was performed in accordance to previously-described pediatric studies [19, 20]. In this study the same-subject crossover design was chosen to reduce the effect of heterogeneity in this patient cohort, so that each patient was his or her own control. Patients were randomly assigned in two groups using block-randomization (SIMV and NAVA groups). The SIMV group, starting in SIMV (PC) PS mode, were switched to NAVA after a washout period. This mode change was performed three times. The NAVA group did so inversely by starting in NAVA and ending in SIMV (PC) PS mode. Each ventilation mode was five minutes in duration. In order to avoid false positive and false negative effects of subsequent sequences due to rapid changes between NAVA and SIMV, washout phases with SIMV (PC) PS were carried out in five-minute intervals after each of the changes of ventilation mode, following the protocol of Lee et al. [20]. Since diaphragmatic activity occasionally decreased during SIMV (PC) PS sequences due to the adaption of pressure support, the planned five minutes for NAVA only started after the reappearance of Eadi signals [21]. Ventilator settings were adjusted to maintain respiratory minute volume for SIMV (PC) PS and NAVA. Ventilator parameters, as tidal volume (V_T_), respiratory frequency and minute volume as well as Eadi were relayed to the EIS device, where they were saved for further analysis. EIS measurements were only taken into account during the ventilation sequences, and not during the washout phases.

### Electrical impedance segmentography

For electrical segmentographic impedance measurements, the Angelie® EIS system (EMS Handelsgesellschaft m.b.H., Korneuburg, Austria) was applied. This system displays the division of electrical impedance of four lung segments. Ten electrodes are applied: five ventrally and five on the dorsal thoracic area. For the alternating current (AC) measurements of impedance, two of the 10 electrodes are placed central to the thorax. These two electrodes form the ventral and dorsal center of the remaining eight electrodes, which are placed on each side of the thorax in the middle of each of the four thoracic quadrants. The central electrodes were placed in the middle of the respective mammillary planes. The remaining electrodes were placed on the medioclavicular planes forming a uniform rectangular X. Ten single electrodes with matching cords had to be placed as described on thoracotomized children in our study group. The single electrodes were plugged in individually, in contrast to the butterfly electrodes, which have only one combined patch cable. For non-thoracotomized children, there was regular placement of the butterfly electrodes (Spes Media Srl, Genoa, Italy), which entails combining the four external and one central electrodes, placed on the ventral and dorsal planes of the thorax. The Angelie^®^ processing unit automatically modulates the electrical current in accordance with electric resistance, which is kept between 10 to 500 µA. The AC works with a frequency of approximately five kHz. Changes of impedance are measured by the other eight electrodes with a sampling frequency of approximately 50 kHz. The processing unit is connected to each electrode. Data are processed onto an image via spectral analysis using high- and low-pass filters, depicting a trend in impedance values.

### Statistics

All statistical analyses were performed with IBM SPSS Statistics Version 27 (IBM Corp., Armonk, NY) and RStudio Version 1.3.1093. (RStudio Team (2020), RStudio Integrated Development for R. RStudio, PBC, Boston, MA). For sample-size calculation, a bilateral p-value of 0.05 and power of 0.8 were provided. In a first attempt with three children, we measured a median ratio between the individual total impedance values of SIMV (PC) PS and NAVA of 0.76 (IQR 0.72–0.83). With this, as a prior expected difference between the ventilation modes, we calculated a needed sample size of n = 15. Due to the short amount of time required (approximately 45 min for each child) and the safe methodology, no more than one child was expected to drop out of the study. Owing to the onset of the SARS-Cov2-pandemic, the study had to be halted prematurely, with a final inclusion group of eight patients. Descriptive statistics were presented, depending on the nature of values, as mean standard deviation (SD), median and percentages. Segmental data exported from the EIS device were processed into variables of total thoracic, horizontal and segmental impedance. Total impedance was calculated as the sum of all four quadrants (upper left and right, and lower left and right). Horizontal impedance was derived by calculating the percentage of the left impedance in relation to the total impedance. Similarly, the percentage of the upper impedance was used as a marker for vertical impedance. The median value of total and segmental electrical impedance for each child and each five-minute ventilation sequence was calculated. A median relative difference of impedance change was generated for each child and for every change of ventilation mode. This included the first change from NAVA1 to SIMV1 until the last change from NAVA2 to SIMV2 (and for the other group starting with SIMV1 until NAVA2) of total, right and left, and upper and lower impedance. A Shapiro–Wilk one-sample test was performed to evaluate the normal distribution of all cumulative and singular parameters. Normally-distributed values were compared by using the Student’s t-test. For comparison of multiple, non-normally distributed variables, the Mann–Whitney U test was performed. To calculate the statistical significance of the relative differences between ventilation modes, a Wilcoxon-test was used. A test variable of “1 “ was applied to account for the null-hypothesis of expecting no difference of the two measured impedances between both ventilatory modes (ratio of one). The computed relative differences of impedance data were compared based on the applied electrodes (single and butterfly electrodes) via a two-sample Wilcoxon-test. Simultaneously recorded ventilation settings during NAVA and SIMV (PC) PS sequences were compared via a two-sample Student’s *t*-test to detect any difference in ventilatory conditions. A one-way analysis of variance (ANOVA) in combination with a Tukey’s post-hoc correction was used to determine the differences of impedance when changing the ventilation from NAVA to SIMV (PC) PS in each child. A p-value of < 0.05 indicated statistical significance.

## Results

Altogether, eight children fulfilled the inclusion criteria for EIS measurements. Demographic data are shown in Table 1.

**Table 1:** Demographic data

n	Age(d)	Sex	Weight(g)	Diagnoses	Reason for admission	MV(d)
1^a^	65	f	5100	Infusothorax	s/p CPR	8
2^a^	104	m	4200	Respir. failure	Hypertrophic CMP	1
3	5	m	3480	Postoperative	Ebstein anomaly	6
4^a^	208	m	6600	Sepsis	Aortic coarctation	16
5	20	m	3100	Postoperative	Fallot tetralogy	6
6^a^	73	m	3900	Postoperative	ASD	1
7^a^	9	f	3500	Respir. failure	MAS	9
8	27	m	3000	Postoperative	Restrictive CMP	10
M (IQR)	46 (12–96)		3700 (3195–4875)			7 (1.75–9.25)

*n* patient identification number, *d* days, *g* gram, *MV* length of mechanical ventilation before the study, *f* female, *s/p* status post, *CPR* cardiopulmonary resuscitation, *m* male, *respir*. Respiratory, *CMP* cardiomyopathy, *ASD* atrioventricular septal defect, *MAS* meconium aspiration syndrome, *M* median, *IQR* interquartile range

^a^Represents children with evaluable results of segmental impedance data

As this was designed as a prospective crossover study, one of two groups, consisting of three children, started with ventilation on NAVA, while five children started on SIMV (PC) PS.

The real-time user interface of the Angelie^®^ EIS system shows the percentage share of distribution of segmental electric impedance and main ventilatory parameters. Individually-measured electric impedance values are given as arbitrary units (a.u.). These measured a.u. showed high variability in total electric impedance (median 435 a.u., IQR 186–1461 a.u.) with a median segmental impedance division of the upper left (UL) segment of 19% (IQR 1–32%), the upper right (UR) segment of 8% (IQR 1–17%), the lower left (LL) segment of 21% (IQR 15–37%) and the lower right (LR) segment of 33% (IQR 14–56%).

By performing a one-sample Shapiro–Wilk test, the distribution of relative difference of impedance, secondary to the ventilation mode, was assessed, whilst a p-value greater than 0.05 was expected to distinguish normal distribution. A normal distribution of data was found in vertical and horizontal impedance, independent of ventilation mode or change during all crossovers (total vertical impedance p = 0.28, total horizontal impedance p = 0.11).

Relative difference of impedance performed with butterfly electrodes showed normal distribution throughout all measurements in total (total p = 0.24, vertical p = 0.90, horizontal p = 0.84) and crossovers. However, when single electrodes were used, all total values of both (p = 0.02) horizontal and vertical impedance (p = 0.03) were not normally distributed (p = 0.001).

**Fig. 1:**
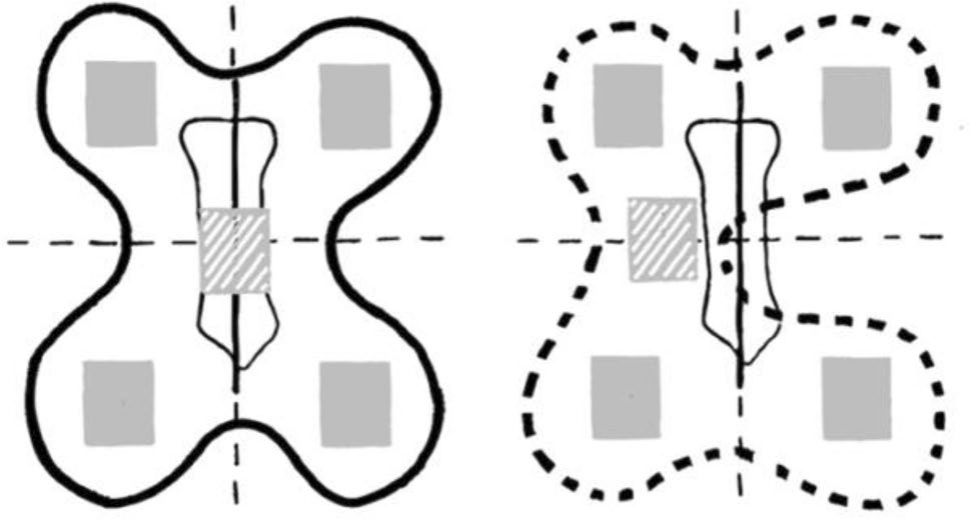
Measuring area of butterfly and single electrodes. On the left, a butterfly electrode placed over the sternum is depicted. The measuring area of single electrodes is depicted on the right. Both depictions are subdivided into the respective four quadrants of measurement. Since single electrodes were used in recently-thoracotomized children, the central electrode was placed on the left side of the thoracotomy site

In three of the eight children, more than one of the segmental impedance values in one or more sequences showed less than four percent of total impedance. As these three children had undergone cardiac surgery, the use of butterfly electrodes was not applicable owing to median thoracotomy. In the remaining five children, only one had been thoracotomized and had to be measured by using single electrodes (Table 1 and Fig. 1 Measuring area of butterfly and single electrodes).

When omitting impedance data of the aforementioned three children, with little or no segmental data, median segmental impedance division amounted to 26% (IQR 22–39%) of the UL, 16% (IQR 5–19%) of the UR, 20% (IQR 15–28%) of the LL and 33% (IQR 15–37%) of the LR segment. A difference of acquired data between single and butterfly electrodes was found when measuring cumulative horizontal impedance (p = 0.05, Table 2).

**Table 2:** Median of the relative differences between all sequences and variables depending on electrode type

		Butterfly electrodes	Single electrodes	p-value
		m (IQR)		
Total impedance	Total	0.75 ± (0.50–1.45)	1.42 ± (0.22–4.49)	0.86
	Change1	0.75 ± (0.51–0.81)	0.23 ± (0.15–3.19)	1.00
	Change2	0.89 ± (0.48–1.88)	1.06 ± (0.13–4.13)	0.29
	Change3	1.12 ± (0.53–2.29)	3.05 ± (1.37–6.65)	0.59
Vertical impedance	Total	0.69 ± (0.55–0.96)	0 ± (0–0.42)	0.77
	Change1	0.69 ± (0.54–0.89)	0 ± (0–1.04)	0.11
	Change2	0.80 ± (0.55–1.43)	0 ± (0–0.35)	0.11
	Change3	0.78 ± (0.55–1.26)	0.13 ± (0–1.51)	1.00
Horizontal impedance	Total	0.90 ± (0.85–1.25)	1.00 ± (0.22–1.30)	0.05
	Change1	0.96 ± (0.76–1.27)	1.08 ± (0.24–1.29)	0.59
	Change2	0.92 ± (0.85–1.41)	1.10 ± (0.22–2.70)	1.00
	Change3	0.87 ± (0.55–1.22)	1.00 ± (0.25–1.17)	0.59

Data are presented as median (interquartile range)

The mean weight of the remaining five children was 4660 ± 1234 g (g). Mean weight of the other three children with limited segmental impedance data was lower in comparison (mean 3193 ± 253 g, p = 0.04). After omitting the data of the three children with limited segmental data, a difference in total transthoracic impedance during the first change of NAVA and SIMV (PC) PS was obtained (median 0.70, IQR 0.36–0.81, p = 0.02). The remaining total, horizontal and vertical data showed no differences in electrical impedance.

**Fig. 2:**
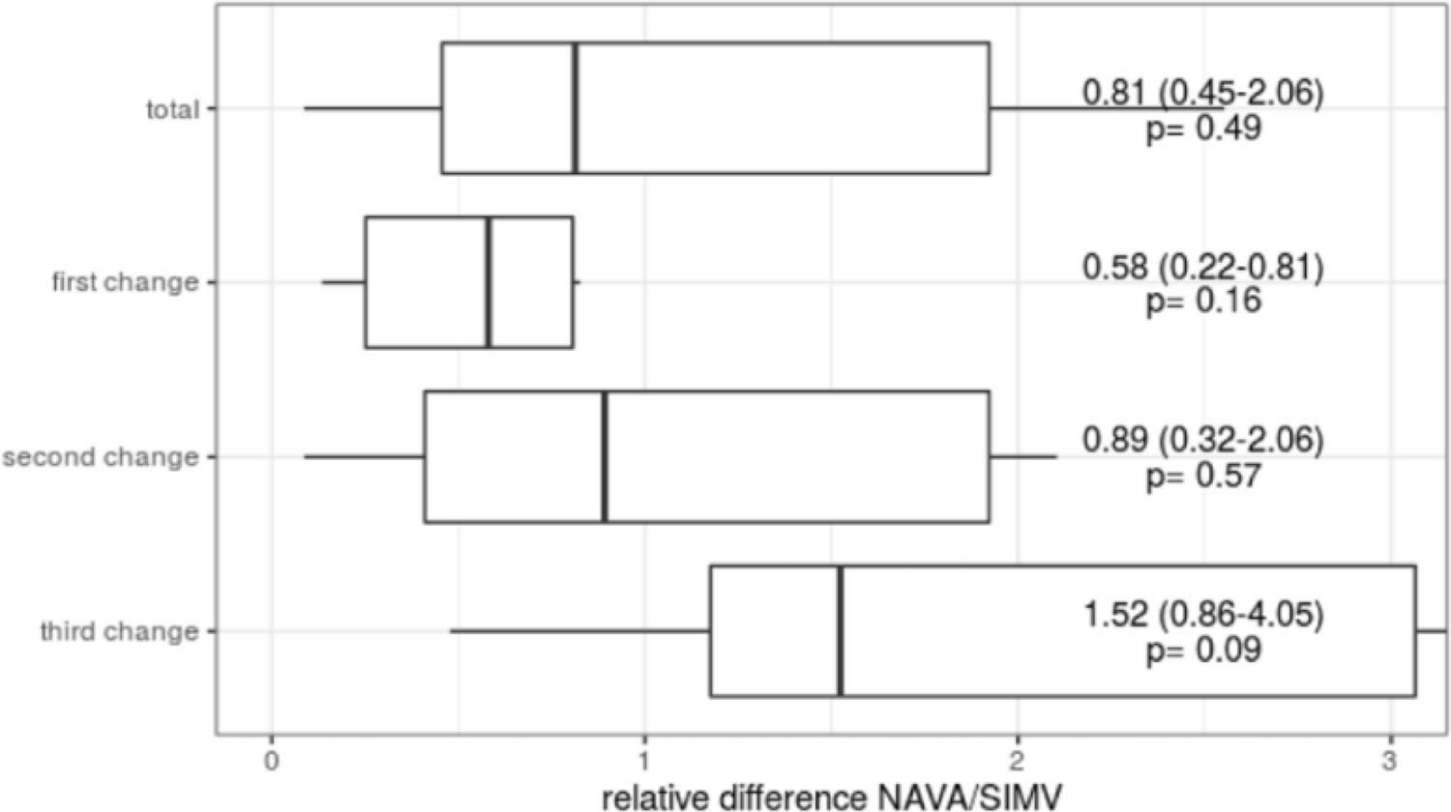
Cumulative and singular total impedance shift depending on the applied breathing method. Median ± IQR of the relative difference between measured impedance during NAVA and SIMV (PC) PS

Data of total impedance showed no differences in terms of change of ventilatory modes, neither after the first, second or third changes between NAVA and SIMV (PC) PS (Fig. 2 Cumulative and singular total impedance shift depending on the applied breathing method.

**Fig. 3:**
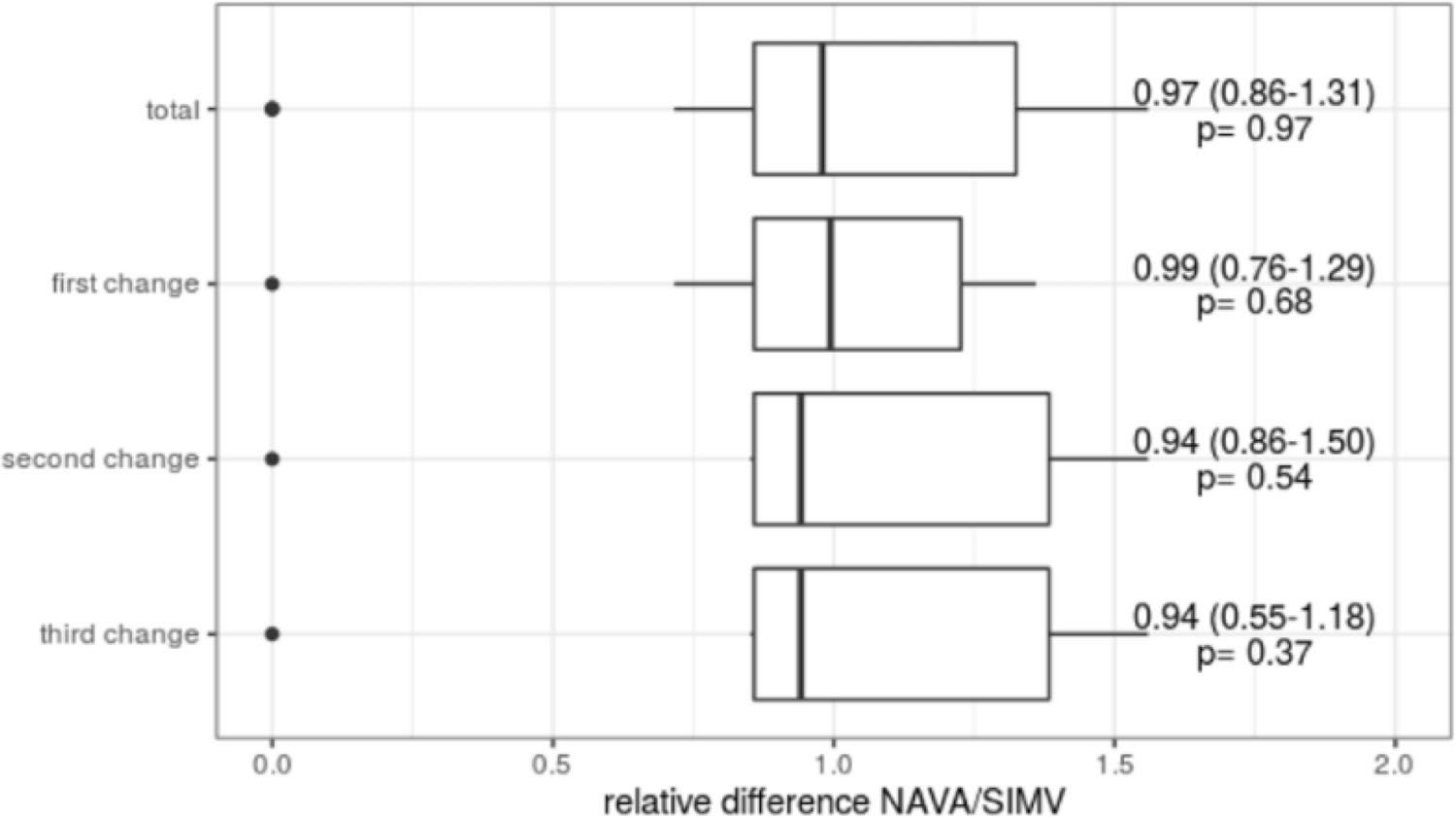
Cumulative and singular impedance shift of the percentage of the left segments depending on the applied ventilatory mode. Median ± IQR of the relative difference between NAVA and SIMV (PC) PS

The observations of horizontal impedance were similar (Fig. 3 Cumulative and singular impedance shift of the percentage of the left segments depending on the applied ventilatory mode.).

**Fig. 4:**
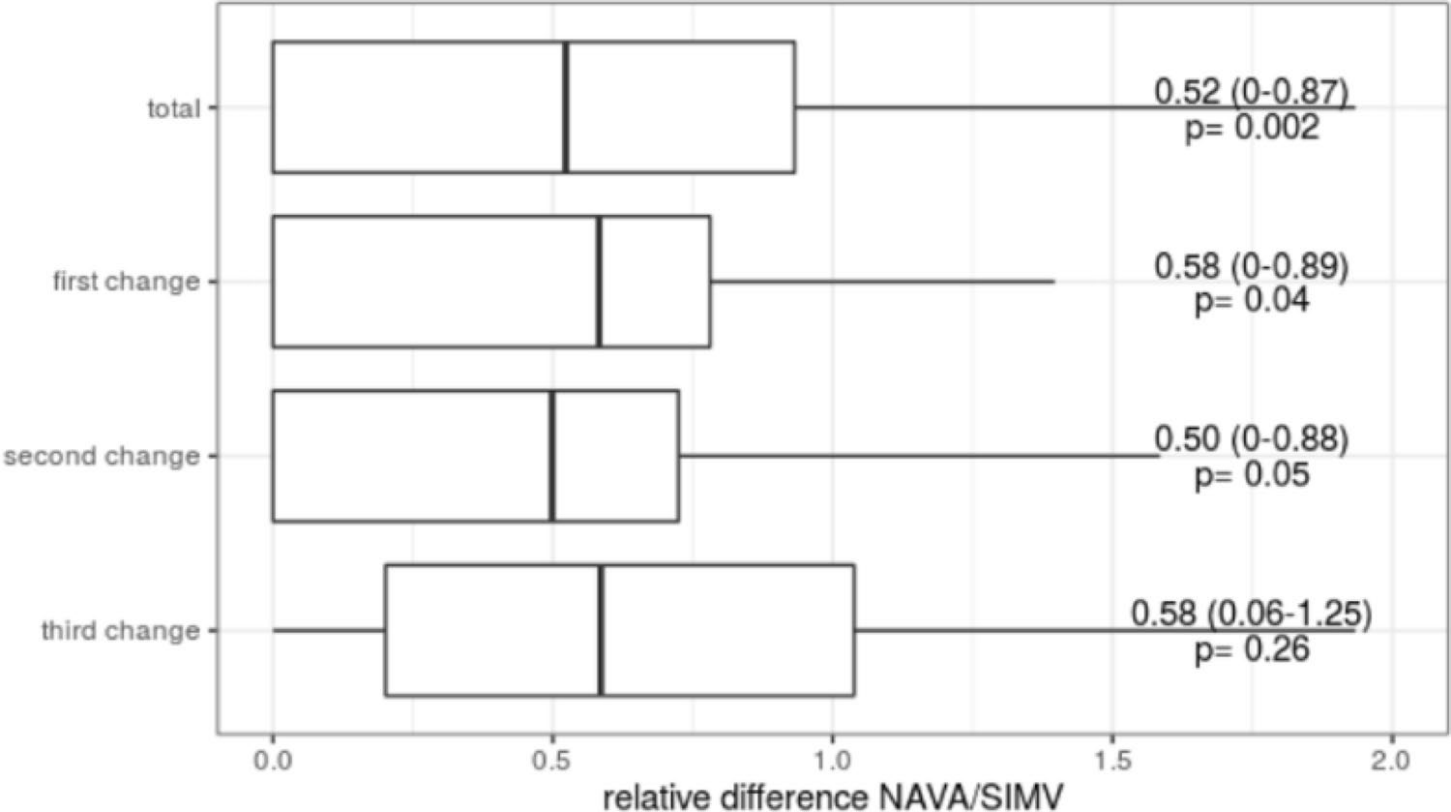
Cumulative and singular impedance shift of the percentage of the upper segments depending on the applied ventilatory mode

A difference of vertical impedance, however, was found both, after the first (0.58 IQR 0–0.89; p = 0.04) and second ventilatory mode changes (0.50 IQR 0–0.88; p = 0.05), as well as of the total impedance (0.52 IQR 0–0.87; p = 0.002, Fig. 4 Cumulative and singular impedance shift of the percentage of the upper segments depending on the applied ventilatory mode).

In comparison, regardless of the first ventilatory mode in this crossover design, no differences in impedance were detected (total impedance, p = 0.68; vertical impedance, p = 0.26; horizontal impedance, p = 0.68).

ANOVA showed no impact of the ventilation mode in the a.u. of total electrical impedance (F(3.28) = 0.4572, p = 0.71). Also the ventilatory mode did not impact the percentage of left impedance (F(3.28) = 0.2849, p = 0.84) or the percentage of upper impedance (F(3.28) = 0.2456, p = 0.86).

The comparison of the ventilation settings between NAVA and SIMV (PC) PS revealed no differences for V_T_ (p = 0.54), frequency (p = 0.207), PEEP (p = 0.18) or minute volume (p = 0.45).

Altogether, a difference in vertical electrical impedance was detected when switching between NAVA and SIMV (PC) PS. This effect was detected in all measured impedances, as well as during the first and second change of ventilation mode.

## Discussion

Performed as a prospective case–control crossover trial of NAVA and SIMV (PC) PS, differences of impedances were assessed by segmentography using the Angelie® device. To date, ventilatory monitoring has been mainly limited to general information and snapshots of the lung or radiation-associated imaging methods without real-time information of regional dynamic lung mechanisms. Therefore, as a bedside tool, the Angelie® segmentography device is simple to implement in children, without causing distress during electrode placement or skin irritation.

As a case–control trial, each child served as her or his own control to reduce interpersonal differences. By further performing a crossover of NAVA and SIMV (PC) PS, potential influences of each initial ventilation mode were presumably diminished. The aim was to assess the detection of this same external intervention by means of electrical impedance. Our study design allowed to conclude that there was a reduction and vertical shift of impedance from transthoracic to lower lung segments during NAVA when compared to SIMV (PC) PS. This effect has shown to be particularly pronounced in NAVA ventilation, by improved patient-ventilator synchronization, which can be attributed to a neurally-driven trigger mechanism [7–10, 22]. Recruitment of dependent lung areas during spontaneous ventilation has been documented by various authors [23, 24]. However, it could also be assumed that this shift of impedance was exaggerated due to the impairment of segmental data in some of the children. When excluding data measured by single electrodes, the aforementioned vertical shift was shown to be less pronounced.

In our analysis, neither V_T_, PEEP nor minute volume differed between NAVA and SIMV (PC) PS. Documented ventilatory settings of this present study, therefore, were comparable to a recent study by Baez Hernandez et al. that reported no change of V_T_ during NAVA ventilation [7]. However, other interventions comparing NAVA and conventional ventilation in pediatric patients have reported decreased PIP levels on NAVA [20, 22, 25], reduced V_T_ in NAVA-ventilated children [20] and increased respiratory rates when compared to pressure-supported ventilation [25].

In our study, the ventilation mode did not appear to impact total electrical impedance. Furthermore, no differences in total, vertical or horizontal impedance were detected, irrespective of whether NAVA or SIMV (PC) PS was the first ventilation mode. Throughout all crossover sequences, no differences in total impedance concerning ventilation modes were observed. However, there was a difference in vertical impedance after the first and second change between NAVA and SIMV (PC) PS.

A recent study utilizing the same EIS monitoring system on healthy, non-sedated and spontaneously-breathing infants reported technical and clinical difficulties in obtaining reliable impedance measurements and experienced a high patient dropout rate of 33% [14]. Children included in our study, however, were all intubated and sedated; hence, individual measurement biases, such as movement, were ruled out.

Nevertheless, impedance segmentography has shown to be a useful tool in spontaneously-breathing four year-olds with bronchopulmonary dysplasia for segmental evaluation after inhalation of salbutamol [26]. Singular segmental impedance data, however, were not consistently measurable in our cohort. Data was particularly limited when measuring the apical sections. In the upper right segments, electrical impedance could only be measured in four of the studied children.

By its crossover design, initial data from the three children with few or no segmental measurements in the calculation of relative differences were included. When omitting these children from the analysis, in whom at least two segments accounted for less than four percent of total impedance, a segmental shift of distribution in impedance was found, similar to the results of Reiterer et al. [14].

In summary, these results, measured by a case–control trial with a crossover of two ventilation modes, revealed that EIS did not appear to reliably measure changes of impedance between NAVA and SIMV (PC) PS, when both, single and butterfly electrodes, are used in one study group. In addition, various studies have also shown a lack of comparison of ventilation modes by different methods [20, 27, 28].

Optimal placement of the electrodes, therefore, should be highlighted since the lack of a segmental impedance measurement was potentially caused by required use of single electrodes in thoracotomized children. The butterfly electrode ensures an equal distance between each of the incorporated electrodes. However, since four of the studied children had previously undergone extensive heart surgeries, only single electrodes could be used. In these patients, the central electrode was placed to the left side of the scar, instead of in the exact center of the four other electrodes as in the butterfly electrodes. Therefore, interference with correct and comparable measurements cannot be ruled out completely as the measuring area appears to be displaced (Fig. 4). On the other hand, it should be mentioned that only one size of butterfly electrode is currently available. Size-adjusted electrodes for different patients would be preferable to increase the accuracy of segmental data. For these reasons, we deemed it necessary to consider and separate these particular patient groups in the data analysis.

By not excluding patients requiring single electrodes to perform measurements, we could show that the application of butterfly electrodes is limited owing to the children’s size and previous thoracic surgeries. Furthermore, this underlines the limitation of using single electrodes due to a potentially altered measuring area.

Also segmentography data obtained by Angelie® could mainly be measured in children weighing more than 3500 g. One reason for this might be the larger amount of lung tissue between segmental electrodes, allowing for a more distinctive differentiation between each sector and minimizing interference.

Studies of EIT have provided highly-reliable impedance data; also in smaller infants [13, 29]. In contrast to EIS, EIT provides impedance changes of the cross-section of the thorax [27], however, it is usually more complex for bedside evaluation in daily clinical routine.

Although our study population was small and in relation to age and weight, as well as the median days of PICU stay inhomogeneous, it should be pointed out that all children underwent the same length of intervention. On the basis of our critically-ill study population, the period of intervention for each child was kept to a minimum. Applied and investigated ventilation techniques, however, are known to be clinically beneficial when patients are ventilated for longer periods [5, 8, 10]. For patients requiring long-term ventilation, EIS may therefore be a useful device for dynamic continuous bedside monitoring for pediatric intensive care patients.

## Limitations

Two main limitations, as already discussed, are to be considered when applying Angelie® for electrical impedance segmentography: The optimal placement of the electrodes, especially if a thoracotomy requires the use of single electrodes and the limited applicability in children weighing less than 3500 g. However, our rather small study population is a limitation. Given the heterogeneity of paediatric intensive care units, we performed a case–control trial, with each patient being her or his own control to reduce the effect of heterogeneity.

## Conclusions

Using Angelie® as an EIS Monitoring tool enables dynamic monitoring for transthoracic impedance during ventilation of critically ill children. Measurements of singular segmental lung areas, were of low reproducibility due to a necessary modified application of the device on thoracotomized children. Immediate benefits of personalized ventilatory strategies can result when using this simple-to-apply bedside tool for measuring lung impedance.

## Data Availability

Not applicable.
